# Squamous Cell Carcinoma of the Toe Secondary to a Traumatic Injury

**DOI:** 10.7759/cureus.48152

**Published:** 2023-11-02

**Authors:** Pallavi Pokharel, Sahar Panah, Ema Mathai, Fawaz Araim, Omar Karim

**Affiliations:** 1 Surgery, St. Agnes Hospital, Baltimore, USA; 2 General Surgery, Ross University School of Medicine, Bridgetown, BRB; 3 Faculty of Medicine, Ross University School of Medicine, Bridgetown, BRB; 4 Vascular Surgery, St. Agnes Hospital, Baltimore, USA

**Keywords:** skin disease/dermatology, toe amputations, sun exposure, squamous cell carcinoma of necrotic toe, squamous cell carcinoma

## Abstract

Cutaneous squamous cell carcinoma-keratoacanthoma (cSCC-KA) is a benign but aggressive neoplasm arising from keratinizing epidermal cells. In this case, we report a 56-year-old man who developed a KA on the right second toe after experiencing a minor trauma to the same region, without osteolysis or nodal invasion. Second toe amputation was performed under sedation. Our case highlights the features of cSCC-KA, its association with trauma, and the importance of early diagnosis and treatment.

## Introduction

Cutaneous squamous cell carcinoma-keratoacanthoma (cSCC-KA) is most commonly found in sun-damaged areas of the skin, such as the head and neck, and only 2.3% of cases involve the lower extremities [1]. Lower extremity neoplasm, induced secondary to cutaneous injury, is rapidly growing. KA lesions commonly present as hyperkeratotic nodules with a crateriform appearance. KAs are practically indistinguishable from invasive cSCC on radiological examination, but they both share similar histopathological findings of epidermal hyperkeratosis and parakeratosis with centralized keratin plugs in addition to the unique presence of intraepithelial microabscesses and eosinophilia in these tumor cells [2]. Depending on localized versus metastasized spread, treatment and plans are tailored to the patient’s presentation. High clinical standards of care and early screening methods are essential to prevent complications, morbidity, and mortality. This study includes a rare case of trauma-induced toe necrosis, found to be squamous cell carcinoma of origin.

## Case presentation

A 56-year-old African American homeless male with a history of human immunodeficiency virus on antiretroviral therapy presented to the emergency department (ED) with increasing right toe pain and bloody discharge. Several months before the presentation, the patient had a traumatic incident when he dropped a piece of granite on his right foot. He was seen at the Veterans Affairs (VA) for the injury and discharged home, but he failed to follow up.

**Figure 1:**
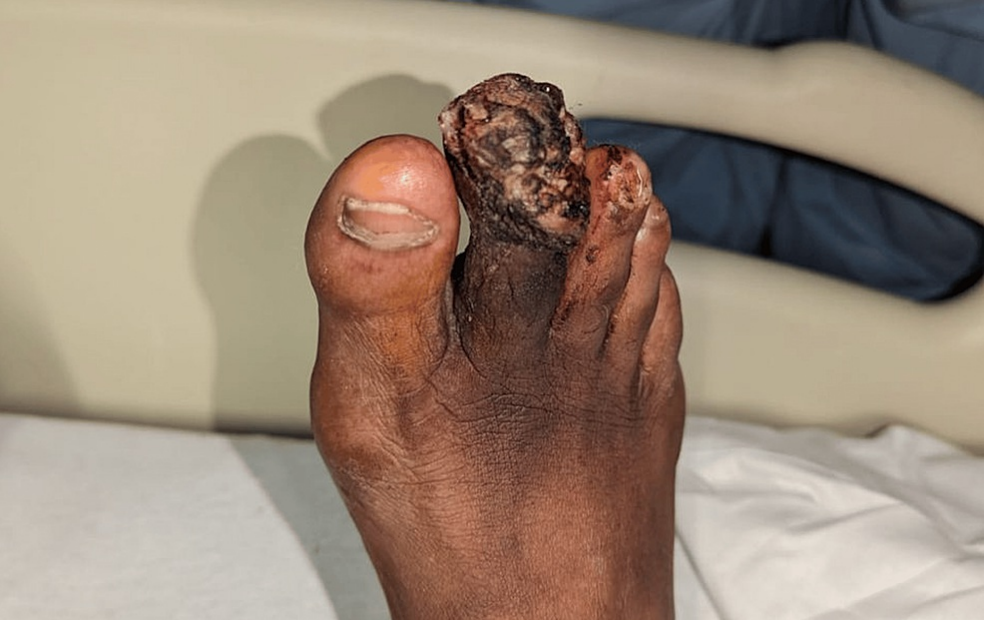
Wet gangrene of the second toe of the right foot.

**Figure 2:**
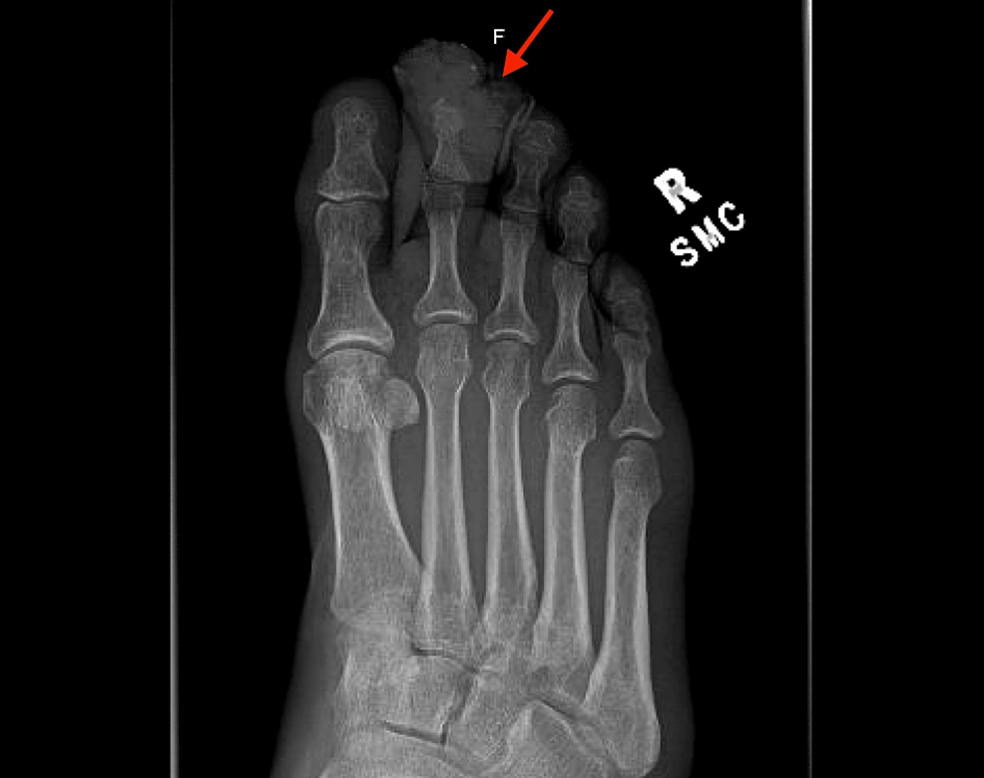
Anteroposterior X-ray of the right foot showing fracture of the second middle phalangeal head and soft tissue swelling.

Upon arrival at the ED, vital signs were within normal limits. Physical examination by an ED physician was notable for wet gangrene of the secondtoe on the right foot and malodorous bloody discharge along with erythema and edema of the surrounding foot (Figure 1). Initial laboratory studies revealed a white blood count (WBC) of 6.2 K/µL and an elevated erythrocyte sedimentation rate of 16 mm/hour. Blood gas analysis showed an elevated lactic acid of 2.0 mmol/L. Other labs were within normal limits. Right foot X-ray showed possible fracture or destruction of the second distal phalanx, a mildly displaced fracture of the second middle phalangeal head, and overlying soft tissue swelling and defect (Figure 2). Because of the unavailability of podiatry at the time, Vascular Surgery was consulted for the management of the necrotic toe and recommended admission.

Physical examination findings were significant for a necrotic right second toe with ischemic discoloration of the forefoot along the second metatarsophalangeal joint region. Swelling and surrounding erythema were noted on the toe along with proximal cellulitis. The toenail was no longer intact. All other toes of the right foot were without discoloration or necrosis. Palpable dorsalis pedis and posterior tibial pulses to the right lower extremity were present.

The patient was reassessed, started on IV antibiotics, and tentatively scheduled for toe amputation the next day. On hospitalization day (HD) three, the patient was taken to the Operating Room for right second toe amputation under sedation. Additionally, a digital block was performed on the right second toe. Circumferential incision was made at the proximal toe and dissection was carried down through soft tissue. The toe was amputated at the interphalangeal joint. The proximal phalanx was debrided with a rongeur. Necrotic tissue was sharply excised. The wound was closed with 3-0 nylon sutures. The incision was irrigated and checked for hemostasis.

The postoperative course was uneventful. The patient remained on IV antibiotics on HD four. During hospitalization, the right foot sutures appeared intact with wound edges well coated. Minimal bleeding was noted at the site with no signs of infection, discoloration, or malodor. Wound care was done with betadine paint and dry sterile dressings, and surgical shoes were provided. The patient was instructed to continue wound care and follow up with vascular surgery and the wound center one to two weeks post-discharge. He was sent home in stable condition with antibiotics on HD five.

Pathology results arrived five days post-discharge and revealed well-differentiated squamous cell carcinoma of the keratoacanthoma type.

## Discussion

KA is an aggressive, rapidly growing skin tumor that is considered a well-differentiated form of cSCC. It has an incidence of 0.6% to 3.0% with a 3:1 male-to-female ratio [1]. Common predisposing risk factors include UV radiation, trauma, chronic inflammation, drugs, tobacco smoke, and immunodeficiency [2]. Nearly 90% of cases are most commonly found in sun-damaged areas of the skin and rarely develop in the lower extremities [3]. KAs in the lower limbs are more challenging to manage due to their unusual anatomic location. They have a higher likelihood of local metastasis up to 30% due to erosion and limited available therapeutic modalities [4]. KA tumors affecting the lower extremities grow rapidly over four to 12 weeks [5]. According to a study by Ghadially et al., 25 out of 238 subjects developed KAs within one week to a year following skin trauma. They established a causal relationship between the destruction of the skin structure and the growth of KAs at the site of injury. However, further studies are needed to define the exact relationship [3]. Compared to traditional KAs, the resolution of tumors in the lower extremities is rare [6]. Early diagnosis and treatment are crucial to preserve foot functionality and avoid amputation [7]. Patients presenting with any inflammatory soft tissue mass in the foot should be assessed using diagnostic imaging and skin incisional biopsy as the gold standard method [6]. Plain imaging is required to evaluate for osteolysis. CT and MRI are useful modalities to assess tumor margins and metastatic lesions throughout the body.

The goal of treatment is to completely remove the tumor and preserve the functionality of the limb. Although there is currently no standardized approach to therapy for KAs of the foot, some studies suggest the use of wide local excision as the first treatment of choice for non-metastatic digital KAs [8]. Amputation is indicated in the most complex cases due to difficult assessment of safety margins or extensive local invasion. Surgical intervention depends on the size and location of the tumor. Surgical intervention is currently considered the gold standard for treatment to avoid recurrence and metastasis [1,2]. In the setting of metastasis to lymph nodes, a biopsy needs to be performed in addition to prophylactic radiation after the complete excision of the primary tumor.

## Conclusions

KA is a benign but aggressive neoplasm that most commonly affects the sun-exposed regions of the skin. Only a few reported cases are presented in the lower extremities. Due to the aggressiveness of KAs of the lower extremities, all patients with chronic non-healing ulcers or any new soft tissue masses in the foot must undergo skin biopsy and diagnostic studies to rule out malignancy and bone invasion. Given the potential risk of local destruction and misdiagnosis in rare cases, most large KAs should be managed definitively to avoid further progression of the disease.
